# Breast Cancer Stage, Surgery, and Survival Statistics for Idaho’s National Breast and Cervical Cancer Early Detection Program Population, 2004–2012

**DOI:** 10.5888/pcd12.140439

**Published:** 2015-03-19

**Authors:** Christopher J. Johnson, Robert Graff, Patti Moran, Charlene Cariou, Susan Bordeaux

**Affiliations:** Author Affiliations: Robert Graff, Patti Moran, Charlene Cariou, Susan Bordeaux, Idaho Department of Health and Welfare, Boise, Idaho.

## Abstract

**Introduction:**

The National Breast and Cervical Cancer Early Detection Program (NBCCEDP) provides access to breast and cervical cancer screening for low-income, uninsured, and underinsured women in all states and US territories. In Idaho, a rural state with very low breast and cervical cancer screening rates, this program is called Women’s Health Check (WHC). The program has been operating continuously since 1997 and served 4,719 enrollees in 2013. The objective of this study was to assess whether disparities existed in cause-specific survival (a net survival measure representing survival of a specified cause of death in the absence of other causes of death) between women screened by WHC and outside WHC and to determine how type of surgery or survival varies with stage at diagnosis.

**Methods:**

WHC data were linked to Idaho’s central cancer registry to compare stage distribution, type of surgery, and cause-specific survival between women with WHC-linked breast cancer and a comparison group of women whose records did not link to the WHC database (nonlinked breast cancer).

**Results:**

WHC-linked breast cancer was significantly more likely to be diagnosed at a later stage of disease than nonlinked breast cancer. Because of differences in stage distribution between WHC-linked and nonlinked breast cancers, overall age-standardized, cause-specific breast cancer survival proportions diverged over time, with a 5.1 percentage-point deficit in survival among WHC-linked cases at 5 years of follow-up (83.9% vs 89.0%). Differences in type of surgery and cause-specific survival were attenuated when controlling for stage.

**Conclusion:**

This study suggests that disparities may exist for Idaho WHC enrollees in the timely diagnosis of breast cancer. To our knowledge, this is the first study to publish comparisons of cause-specific breast cancer survival between NBCCEDP-linked and nonlinked cases.

## Introduction

The Breast and Cervical Cancer Mortality Prevention Act of 1990 (Public Law 101–354) directed the Centers for Disease Control and Prevention (CDC) to create the National Breast and Cervical Cancer Early Detection Program (NBCCEDP), which increases access to breast and cervical cancer screening and detection services for low income, uninsured, and underinsured women (1,2). Women’s Health Check (WHC), Idaho’s breast and cervical cancer early detection program, is funded through NBCCEDP and state legislative appropriations (3).

Idaho’s WHC program primarily serves women who are US citizens or noncitizens who have lived in the United States for 5 or more years, have incomes up to 200% of the federal poverty guidelines, and have no health insurance coverage for Papanicolaou (Pap) tests or mammograms (3). WHC provides Pap tests for women aged 40 to 64 years every 3 years, or every 5 years with a human papillomavirus (HPV) co-test; it provides an annual screening mammogram for women aged 50 to 64 years, plus a clinical breast examination. In addition, WHC offers limited enrollment and screening for women who meet income and insurance status requirements beginning at age 30 if they are symptomatic for breast or cervical cancer. Women older than 65 who are not eligible for Medicare or cannot afford Medicare Part B are also eligible for WHC screening. The WHC program has operated continuously since 1997, and during fiscal year 2013 it had 4,719 women enrolled in the program (3). Women with breast or cervical cancer or precancerous conditions who were enrolled in WHC before a tissue biopsy diagnosis can qualify for treatment through the Idaho Medicaid Breast and Cervical Cancer Program. 

The Cancer Data Registry of Idaho (CDRI) is a population-based cancer registry that collects incidence and survival data on all cancer patients who reside in the state of Idaho or who are diagnosed or treated for cancer in the state (4). CDRI meets program standards of the National Program of Cancer Registries and is recognized as a “gold standard” registry for data quality, completeness, and timeliness as designated by the North American Association of Central Cancer Registries (5,6). Since 1999, CDRI has linked with the WHC database to identify potentially missed breast cancers in the CDRI database, reconcile differences between the 2 systems, and update appropriate data fields to capture post-linkage information (7). This study compared stage distribution, type of surgery, and survival between women with WHC-linked breast cancer diagnosed during the period 2004–2012 and a comparison group of women whose records did not link to the WHC database (nonlinked breast cancer).The study followed comparisons between NBCCEDP-linked breast cancer cases and nonlinked breast cancer cases on stage at diagnosis (8–12), treatment (9,11–13), and mortality (12).Survival rates are a crucial metric for evaluating advances in treatment and measuring differences in outcomes between populations; however, net measures of cancer survival are lacking for women diagnosed through the NBCCEDP. The objective of this study was to assess whether disparities exist in cause-specific survival (a net survival measure representing survival of a specified cause of death in the absence of other causes of death) between women with WHC-linked and women with nonlinked breast cancers and to understand how differences in type of surgery or survival are related to stage at diagnosis. To our knowledge, this is the first study to publish comparisons of cause-specific breast cancer survival between women with NBCCEDP-linked and women with nonlinked cases.

## Methods

WHC uses CaST software (Information Management Services, Inc) provided by CDC to NBCCEDP grantees for data collection, database management, and reporting. Details of CDRI’s data collection procedures and coding rule use were previously reported (4). At least once per year since 1999, data have been linked between WHC and CDRI databases by using probabilistic linkage software, currently Link Plus, version 2.0 (CDC) (7). This study used CDRI cases diagnosed from 2004 through 2012 that were linked to WHC in November 2013. Because this project classified as program evaluation by the Idaho Department of Health and Welfare Research Determination Committee, it was exempt from institutional review board review.

This study was limited to Idaho resident women aged 30 to 64 years who were diagnosed with breast cancer from 2004 through 2012 identified from the CDRI database, who were linked to the WHC database (WHC-linked) and a comparison group of women with the same date and age at diagnosis criteria whose records did not link to the WHC database (nonlinked). Breast cancers reported solely by death certificates were excluded from analyses of stage, treatment type, and survival. Type of breast surgery included breast-conserving surgery (Facility Oncology Registry Data Standards [FORDS] codes 20–24) and mastectomy (FORDS codes 30–80) (14).

Statistical analyses were conducted using SEER*Stat version 8.1.5 (Information Management Services, Inc) and SAS version 9.3 (SAS Institute Inc). Pearson χ^2^ statistics were used to test associations between cross-frequencies of variables, and Cochran–Mantel–Haenszel statistics were used in the analysis of stratified categorical data. We used *t* tests to compare differences between mean ages. *P* values less than .05 were considered to be significant. For analyses using stage distribution as the outcome variable, cancers with unknown stage at diagnosis were excluded from statistical tests. For analyses using type of surgery as the outcome variable, cancers of unknown stage or with associated surgery that was either unspecified or unknown were excluded from statistical tests.

The survival statistics in this study were not designed to be representative of death rates among women with breast cancer, which would also include other causes of death, but rather to facilitate the comparison of WHC-linked and nonlinked groups in terms of death from breast cancer. To ascertain deaths related to a specific cancer site (ie, breast cancer) but not coded to the underlying cause of death, we used the Surveillance, Epidemiology and End Results (SEER) cause-specific death classification to account for causes of death in conjunction with tumor sequence (ie, only 1 tumor or the first of subsequent tumors), site of the original cancer diagnosis, and comorbidities (15). Cause-specific survival was calculated by using the actuarial method for women diagnosed with invasive breast cancer from 2004 through 2010, with breast cancer as the first or only primary cancer, who were aged 30 to 64 years at the time of diagnosis. The end of the study period for survival analyses was set at December 31, 2011, because that is the latest date for which death data were complete. At the time of this study, CDRI had linked all 2004–2011 cancers to state death records from 2004 through 2012 and had conducted National Death Index searches for 2004 through 2011 (16). Women who were not known to be deceased as of December 31, 2011, were presumed alive on this date for survival calculations (17). Survival estimates were age-standardized to the International Cancer Survival Standard using the age groups 30 to 39 years, 40 to 49, and 50 to 64 (18).

## Results

From 2004 through 2012, a total of 5,606 in situ and invasive breast cancers were diagnosed among Idaho resident women aged 30 to 64 years and were used in the linkage to the WHC database. Of these, 540 (9.6%) breast cancers linked to WHC. Nine breast cancers were reported solely by death certificates, none of which linked to WHC; these were excluded from analyses of stage, type of surgery, and survival. No cancer record had missing information on age at diagnosis. Thirty-nine records were missing stage information (3 among WHC-linked cancers and 36 among nonlinked cancers) and were excluded from analyses of stage and type of surgery. Twelve records were missing information on type of surgery (0 among WHC-linked cancers and 12 among nonlinked cancers) and were excluded from analysis of surgery type.

The age distribution of the WHC-linked cancers was significantly different from the nonlinked cancers (*P* = .02); women with WHC-linked cancers were slightly younger (*P* = .01 [Table 1]). The mean ages of women in the 2 groups were less than 1 year apart. Women with WHC-linked cancers were significantly more likely to be diagnosed at a later stage than women with nonlinked cancers (43.4% vs 31.3% at regional or distant stage, *P* < .001), even after controlling for age differences between the 2 groups. In age groups for which population-based breast cancer screening is often recommended (40–49 y and 50–64 y), the WHC-linked cancers were significantly more likely to be diagnosed at a later stage (40–49 y, 45.4% vs 33.7%, *P* = .003; 50–64 y, 40.9% vs 29.1%, *P* < .001). Among women 30 to 39 years, for whom breast cancer screening is not recommended, there was no significant relationship between WHC linkage status and stage (*P* = .14).

**Table 1:** Breast Cancers in Idaho Resident Women (In Situ and Invasive) by WHC Linkage Status and Age and Stage at Diagnosis, 2004–2012

Age and Cancer Stage at Diagnosis	WHC-Linked Cancer^a^	Nonlinked Cancer^a^	*P* Value^b^
**5-year age group, y**			
30–34	18 (3.3)	82 (1.6)	.02
35–39	16 (3.0)	211 (4.2)	
40–44	62 (11.5)	487 (9.6)	
45–49	101 (18.7)	861 (17.0)	
50–54	113 (20.9)	1,039 (20.5)	
55–59	116 (21.5)	1,154 (22.8)	
60–64	114 (21.1)	1,232 (24.3)	
Mean age, y	52.0	52.9	.01
**All ages**			
In situ or localized	304 (56.6)	3,451 (68.7)	<.001
Regional or distant	233 (43.4)	1,570 (31.3)	
**Aged 30–39 y**			
In situ or localized	14 (41.2)	158 (54.5)	.14
Regional or distant	20 (58.8)	132 (45.5)	
**Aged 40–49 y**			
In situ or localized	89 (54.6)	885 (66.3)	.003
Regional or distant	74 (45.4)	450 (33.7)	
**Aged 50–64 y**			
In situ or localized	201 (59.1)	2,408 (70.9)	<.001
Regional or distant	139 (40.9)	988 (29.1)	

Abbreviation: WHC, Women’s Health Check.

a Values are N (%) unless otherwise indicated.

b Pearson χ^2^ statistics were used to test associations between cross-frequencies of variables, and *t* tests were used to compare differences between mean ages. *P* values <.05 were considered to be significant. Cancers of unknown stage were excluded from tabulations and statistical tests.

Table 2 presents first course of surgery by stage at diagnosis and WHC linkage status. Overall, women with WHC-linked cancers were significantly less likely to have breast-conserving surgery (52.3% vs 59.0%, *P* = .01), but this relationship was attenuated when adjusting for stage (*P* = .36). Stratified by stage, there was no significant relationship between type of surgery and WHC linkage status (*P* = .34 for in situ, *P* = .34 for localized, *P* = .61 for regional, and *P* = .34 for distant).

**Table 2:** Type of Surgery by WHC Linkage Status and Stage at Diagnosis, Breast Cancer in Idaho Resident Women, 2004–2012

Cancer Stage at Diagnosis and Type of Surgery	WHC-Linked Cancers, N (%)^a^	Nonlinked Cancers, N (%)^a^	*P* Value^b^
**All stages**			
No surgery	36 (6.7)	269 (5.4)	.01
Breast conserving surgery	281 (52.3)	2,954 (59.0)	
Mastectomy	220 (41.0)	1,786 (35.7)	
**In situ**			
No surgery	5 (7.4)	46 (4.4)	.34
Breast conserving surgery	48 (70.6)	707 (67.5)	
Mastectomy	15 (22.1)	295 (28.1)	
**Localized**			
No surgery	5 (2.1)	46 (1.9)	.34
Breast conserving surgery	150 (63.6)	1,634 (68.2)	
Mastectomy	81 (34.3)	715 (29.9)	
**Regional**			
No surgery	9 (4.6)	65 (4.7)	.61
Breast conserving surgery	77 (39.3)	590 (42.9)	
Mastectomy	110 (56.1)	721 (52.4)	
**Distant**			
No surgery	17 (45.9)	112 (58.9)	.34
Breast conserving surgery	6 (16.2)	23 (12.1)	
Mastectomy	14 (37.8)	55 (28.9)	

Abbreviations: WHC, Women’s Health Check.

a Percentages may not sum to 100% because of rounding.

b Pearson χ^2^statistics were used to test associations between cross-frequencies of variables. *P* values <.05 were considered to be significant. Cancers with unknown stage or unspecified or unknown surgery were excluded from tabulations and statistical tests.

By stage at diagnosis, there were no significant differences in age-standardized, cause-specific breast cancer survival proportions between women with WHC-linked cancers and women with nonlinked cancers at 1 to 5 years of follow-up (Table 3). However, because of differences in stage distribution between WHC-linked and nonlinked cancers, overall age-standardized, cause-specific breast cancer survival proportions diverged over time, with a 5.1 percentage-point deficit in survival among women with WHC-linked cancers at 5 years of follow-up (83.9% vs 89.0%).

**Table 3:** Age-Standardized, Cause-Specific Breast Cancer^a^ Survival Proportions by WHC Linkage Status and Stage at Diagnosis, Idaho Resident Women, 2004–2010^b^

Cancer Stage at Diagnosis	Survival Time, y	Age-standardized Cause-Specific Survival, WHC-Linked Cancers^b^		Age-standardized Cause-Specific Survival, Nonlinked Cancers^b^	
		N	% (95% CI)^c^	N	% (95% CI)^c^
Total, all stages^d^	1	305	98.6 (96.1–99.5)	2,685	98.6 (98.1–99.0)
	2		94.0 (90.3–96.4)		95.9 (95.1–96.6)
	3		91.9 (87.8–94.7)		93.5 (92.4–94.4)
	4		88.1 (82.8–91.9)		90.7 (89.3–91.9)
	5		83.9 (77.1–88.9)		89.0 (87.5–90.4)

Localized	1	147	100.0 (NC)	1,566	99.7 (99.2–99.9)
	2		98.4 (94.6–99.5)		98.9 (98.2–99.3)
	3		98.4 (94.6–99.5)		97.9 (97.0–98.6)
	4		97.7 (93.6–99.2)		96.7 (95.5–97.6)
	5		95.5 (87.3–98.5)		96.3 (94.9–97.3)

Regional	1	132	100.0 (NC)	973	99.2 (98.3–99.6
	2		93.7 (86.6–97.1)		95.1 (93.4–96.4)
	3		88.7 (80.8–93.5)		92.1 (89.9–93.8)
	4		85.0 (75.2–91.2)		88.1 (85.5–90.3)
	5		80.2 (68.3–88.0)		85.4 (82.4–87.9)

Distant	1	24	83.8 (62.5–93.5)	121	84.6 (76.8–89.9)
	2		69.7 (47.2–84.1)		67.6 (58.2–75.3)
	3		69.7 (47.2–84.1)		51.5 (41.8–60.4)
	4		51.0 (29.4–69.0)		40.4 (30.8–49.8)
	5		39.4 (17.0–61.3)		37.8 (28.2–47.3)

Abbreviations: CI, confidence interval; NC, could not be calculated; WHC, Women’s Health Check.

a Cause-specific breast cancer survival is a net survival measure representing survival of a specified cause of death in the absence of other causes of death.

b First primary cases with follow-up through December 2011. Survival calculations use actuarial method. Age standardized to the International Cancer Survival Standard 1, with age groups 30–39 y, 40–49 y, 50–64 y (18).

c 95% CIs use complementary log–log transformation.

d Total includes cases with unknown stage.

## Discussion

The Idaho WHC program began in 1997, with a focus on targeting medically underserved and symptomatic women for screening for breast and cervical cancers. Consistent with national NBCCEDP estimates (1), fewer than 15% of Idaho’s WHC-eligible women are currently served by the program. In 2012, an estimated 246,645 women in Idaho were aged 40 to 64 years; 69,566 had incomes below 200% of federal poverty guidelines, of whom 28,484 (40.9%) were uninsured (19). Some additional women had health insurance but were uninsured for breast or cervical cancer screening. The percentage of Idaho women who had incomes below 200% of the federal poverty guidelines and were uninsured differed by age: 13.4% of women aged 40 to 49 years and 10.4% of women aged 50 to 64 years (19). Younger women were somewhat overrepresented in the WHC program, as illustrated by the younger age distribution of the WHC-linked breast cancers compared with nonlinked cancers.

Age-appropriate screening for breast and cervical cancers has helped reduce death rates from these diseases. The US Preventive Services Task Force currently recommends biennial screening mammography for women aged 50 to 74 years (20). In 2012, Idaho ranked 50th among states and the District of Columbia in the proportion of women aged 40 years or older and aged 50 to 74 who had received a screening mammogram for breast cancer within the previous 2 years (21). From 2008 through 2012, WHC provided mammograms to 10,112 women and Pap tests to 8,491 women (22). However, since the WHC program began in Idaho, overall screening trends for breast and cervical cancer have essentially remained flat (21). Because state programs under the NBCCEDP cover relatively small proportions of the total age-appropriate screening populations, the signal related to these programs may be lost in the noise inherent to population-based surveys.

Despite Idaho’s low cancer screening rates, which may act to minimize differences, this study was able to detect significant differences between WHC-linked breast cancers and nonlinked breast cancers. A main finding of this study is that from 2004 through 2012, among women of ages for which breast cancer screening is recommended, WHC-linked cases of breast cancer were significantly more likely than nonlinked cancers to be diagnosed at a later stage of disease. This outcome has not been consistently found in comparable studies of state breast and cervical cancer programs. As in Idaho, breast cancers among NBCCEDP enrollees in Florida and Nebraska were significantly more likely to be diagnosed at a later stage (8,12). However, 2 studies of the equivalent program in Massachusetts found that stage at diagnosis of program participants did not differ significantly from comparison groups (9,10). The differences in these results by state may be related to population screening prevalence. In 2012, likely because of the effect of state-based health care reform efforts, Massachusetts had the highest rate of mammography screening in the United States (21). Florida and Nebraska, like Idaho, were below the national median.

We speculate that 1 reason for the increased likelihood of late-stage breast cancer diagnosis is that WHC is not only a screening program but also serves women who have symptoms suspicious of breast or cervical cancer as confirmed by a health care professional. Other states have noted that the majority of women coming into the NBCCEDP program and being screened entered the program because they were symptomatic (8). Of all invasive cancers detected in the Idaho WHC program from 2009 through 2012, one-third were diagnosed because of referral into the program because of abnormal screening results outside the program, and almost another one-third were diagnosed among women whose initial WHC mammogram was for evaluation of symptoms (WHC, unpublished data). In the general population, a higher proportion of breast cancer diagnoses may result from screening of asymptomatic women in the age group 40 to 64 and hence have been detected at an earlier stage. Other reasons why WHC participants in Idaho have an increased likelihood of late-stage breast cancer may be differences in tumor biology, delayed diagnosis, and higher rates of over-diagnosis among nonparticipants (23).

Other studies have shown that information on type of surgery in cancer registries is nearly complete, whereas data on chemotherapy and radiation therapy may be incomplete (24). The surgical treatment data in this study were nearly complete and were comparable to data from the SEER program (25). In SEER registries, the percentage of female breast cancers with no surgery coded or unknown surgery codes was less than 4% for in situ and localized stage and less than 3% for regional stage. Among Idaho breast and cervical cancers in this study, less than 5% of in situ and regional breast cancers and less than 3% of localized cancers had no surgery coded or unknown surgery codes.

Women diagnosed with early-stage invasive breast cancer in the United States typically have either mastectomy or breast-conserving surgery in combination with radiation therapy. Both options are equally effective in terms of survival (26). In this study, we found a significant difference in 5-year cause-specific survival from breast cancer between WHC-linked cancers and nonlinked cancers but not when stratified by stage at diagnosis. The overall difference in survival may be related to potential lead-time bias among the nonlinked cases. There may have been an increase in the time from diagnosis to survival outcome because the preclinical detection time was included in survival calculations, but there may be no change in the type or date of the outcome. The stage-specific survival results suggest that outcomes were similar for WHC-linked and nonlinked cases after diagnosis. As with our cause-specific survival results stratified by stage, Bhuyan et al used logistic regression in Nebraska to model all-cause deaths at 1 and 5 years after breast cancer diagnosis and found the adjusted odds of death did not differ significantly between NBCCEDP cancers and non-NBCCEDP cancers (12).

Our results indicate that women with WHC-linked breast cancer appeared to receive the same level of care as those with nonlinked cancers. For both WHC-linked and nonlinked cancers, about 80% of women were treated in Idaho hospitals accredited by the American College of Surgeons Commission on Cancer (27). Twenty-five surgeons in Idaho performed about 70% of all breast cancer surgeries in the study population, both for WHC-linked cases (68%) and nonlinked cases (70%). For individual surgeons, the proportion of cases that were WHC-linked differed depending on the percentage of their services that were covered by Medicaid. Differences in type of surgery and survival observed in this study were probably the result of differences in stage at diagnosis. Adjusting for stage, we found no significant differences between WHC-linked and nonlinked cancers in type of surgery or breast cancer cause-specific survival. Again, differences in stage distribution between WHC-linked and nonlinked cancers should not necessarily be construed as a health care disparity because WHC is not strictly a screening program.

Limitations of the routinely collected surveillance data used in this study include a lack of detailed information on women that may have influenced treatment planned or received. Information was not available on pregnancy status, family history, prior medical history, or tumor margins. In addition, the study did not include a review of hormone receptor status, biomarkers, genetic testing results, or presence of other disorders. Because of concerns about data quality, neither radiation therapy nor chemotherapy regimens were reported in this study (24). Another potential limitation is the relatively small number of linked cases, which resulted in limited power to detect differences between WHC-linked cancers and nonlinked cancers. Strengths of this study include use of high-quality data from CDRI and WHC with a low proportion of unknown or missing values for the variables studied and a long history of successful linkages between the 2 databases.

A potential limitation of our study is that the comparison group should have more closely reflected the socioeconomic status (SES) of the WHC-linked cases. Individual-level measures of SES are not collected by central cancer registries and are thus not available for analysis. For the data set used in this study, a census tract-based SES measure of the percentage of the population with incomes below federal poverty guidelines was available for cancers diagnosed from 2004 through 2011. The distributions of stage at diagnosis and type of surgery did not differ significantly by area-based SES. Likewise, stage-specific survival did not differ significantly by area-based SES. Hence, additional results were not reported by area-based SES.

This is the first study to show a breast cancer survival disparity for the NBCCEDP population. Because of differences in stage at diagnosis, this study found that women with WHC-linked cancers were significantly less likely to have breast-conserving surgery and significantly more likely to die of their cancer within 5 years of diagnosis. The findings suggest that WHC is working as intended by focusing on symptomatic or rarely or never screened women who are at greater risk for late-stage cancers. The Idaho Division of Public Health will use these results to attempt to increase the number of new clients who are rarely or never screened, focusing on the reservoir of undiagnosed prevalent cancers. These findings reinforce the necessity of screening women from our priority population given the likelihood of later stage disease and lower survival rates among WHC-linked cancers. These findings will help inform future outreach efforts as well as provide a metric for future evaluation of screening in our priority population. Findings also reinforce the importance of patient outreach, education, and navigation. Potential strategies for maximizing the efficacy of the WHC program include biennial versus annual screening intervals for mammograms and securing nonfederal funding with flexibility in resource allocation. Such funding could be used to perform outreach and effect system changes, such as innovative data collection and increased use of evidence-based strategies in all aspects of WHC program implementation, including patient reminders, one-on-one counseling, tailored small-media outreach, and better use of community–clinical linkages to increase enrollment.

This study suggests that health care disparities may exist for Idaho WHC enrollees in the timely diagnosis of breast cancer and that these disparities drive overall differences in type of surgery and cause-specific survival. This study also illustrates the power of linkages between central cancer registries and NBCCEDP databases for quality improvement, program assessment, and monitoring population health. Future studies should attempt to identify the reasons for the identified breast cancer disparities. Methods for doing so may include surveys of NBCCEDP participants to assess delayed diagnosis and patterns-of-care studies that include additional patient characteristics, tumor biology, and treatment data. The WHC program is important because of Idaho’s very low breast and cervical cancer screening rates.
